# Supplementary material for: Gasdermin D pores are dynamically regulated by local phosphoinositide circuitry
Source: Nat Commun. 2022 Jan 10;13:52. doi: 10.1038/s41467-021-27692-9 (PMC8748731; doi:10.1038/s41467-021-27692-9)
Supplement: Supplementary file 1 — Supplementary Information [file 41467_2021_27692_MOESM1_ESM.pdf]

Gasdermin D Pores Are Dynamically Regulated By Local Phosphoinositide Circuitry

Santa Cruz Garcia et al.

Supplementary Figures and Legends

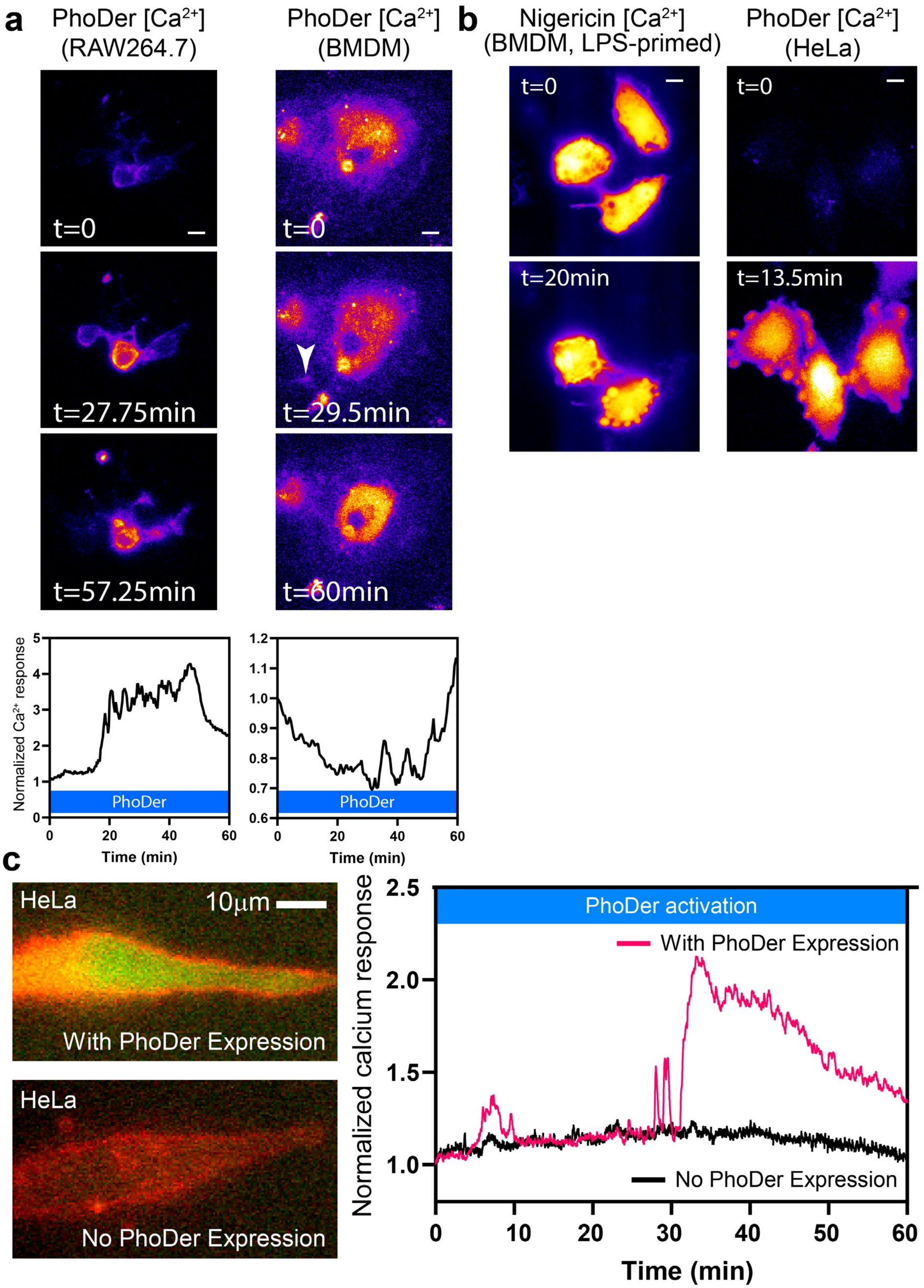
a
PhoDer [Ca2+] (RAW264.7)
PhoDer [Ca2+] (BMDM)
t=0
t=27.75min
t=57.25min
t=0
t=29.5min
t=60min
b
Nigericin [Ca2+] (BMDM, LPS-primed)
PhoDer [Ca2+] (HeLa)
t=0
t=20min
t=0
t=13.5min
Normalized Ca2+ response
PhoDer
Time (min)
c
HeLa
10μm
With PhoDer Expression
HeLa
No PhoDer Expression
PhoDer activation
With PhoDer Expression
No PhoDer Expression
Normalized calcium response
Time (min)

Supplementary Figure 1. PhoDer can be utilized to tune pyroptosis and thus kill cells or drive calcium progression. (a) Appropriate PhoDer activation reproduces the calcium flare response progression in both RAW264.7 macrophage cell line and mouse bone-marrow derived macrophages (BMDM). Arrowhead indicates the location of spontaneous calcium flare; images representative of n=5 (RAW264.7) and n=3 (BMDM) independent cells; scale bars: 10 µm. (b) Strong PhoDer activation can reproduce the "bubble" cell lysis phenotype observed during nigericin-induced inflammatory activation of bone-marrow derived macrophages (BMDM). Note that LPS-primed BMDM has high steady-state calcium level after 4hr LPS-priming, prior to nigericin stimulation, whereas PhoDer activation in HeLa cells began with low intracellular calcium. PhoDer images are representative of n=28 independent cells; scale bars: 10 µm. (c) HeLa cells in the same field-of-view show either GSDMD-activated calcium response or no calcium response at all (right side of panel), depending on the presence of PhoDer; lack of PhoDer expression (green color, left side of panel) prevents the any calcium responses despite membrane calcium biosensor expression (red color). Pseudocolor images are scaled exactly the same way.

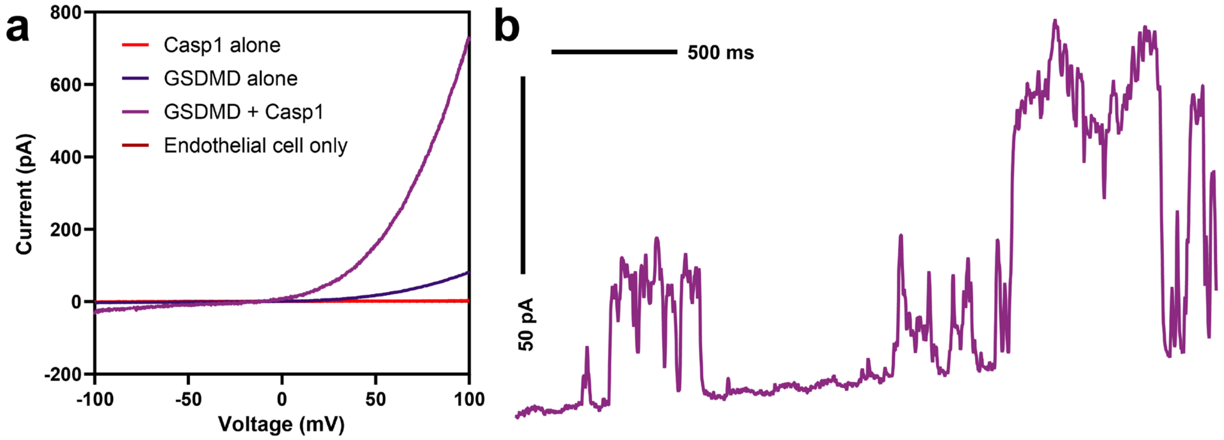

Supplementary Figure 2. Endothelial cells patching also show dynamic open/closing events just as bilayer models and PhoDer activation. (a) Averaged ramp cycles (n=15 cells for each condition) I/V graphs showing the outward rectification of GSDMD pores in living lung endothelial cells during whole cell patch ramp; controls with cell only and caspase 1 alone did not show any current response; recombinant GSDMD protein alone showed slight activation due to limited basal endogenous activity of caspase 1 in these cells. The apparent rectification from these whole-cell patch experiments should not be over-interpreted and does not show an intrinsic pore property. (b) Representative trace for live whole lung endothelial cell single pore events after the injection of mixed recombinant GSDMD and caspase 1.

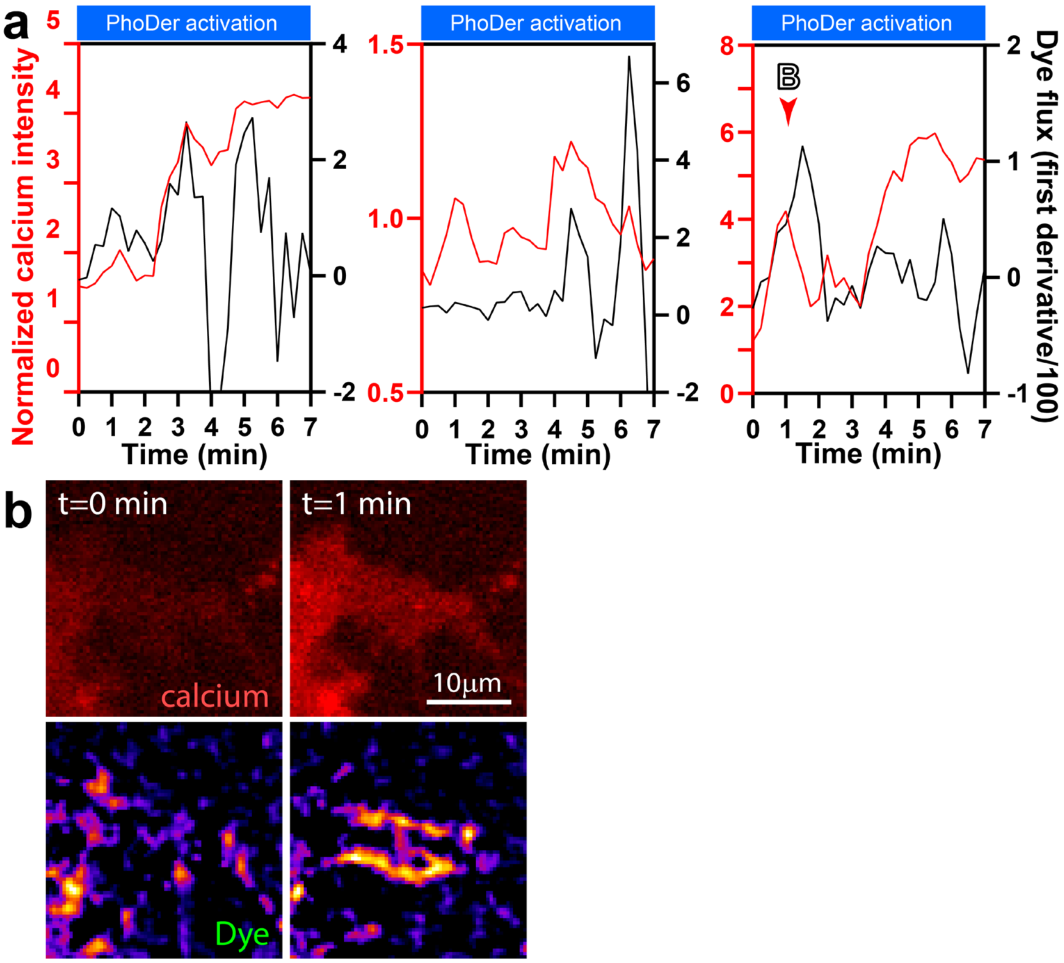

Supplementary Figure 3. Transient flux of a large dye molecule can be spatiotemporally detected in coincidence with optogenetically activated GSDMD-induced calcium flares. (a) Representative calcium traces (red) and corresponding fluorogenic dye flux traces (black) captured from the same regions of interest, in each demonstrating coincidence of calcium flares and flux of the large molecule (time-dependent first-order differential, Methods in Supplementary Information; 3 examples were taken from 3 separate cells. (b) Representative images of the coincident flare event indicated in A) via red arrowhead; the membrane dye flux increase is clearly local in both space and time. Pseudocolor images were adjusted to the same contrast for comparison. Scale bar: 10 μm.

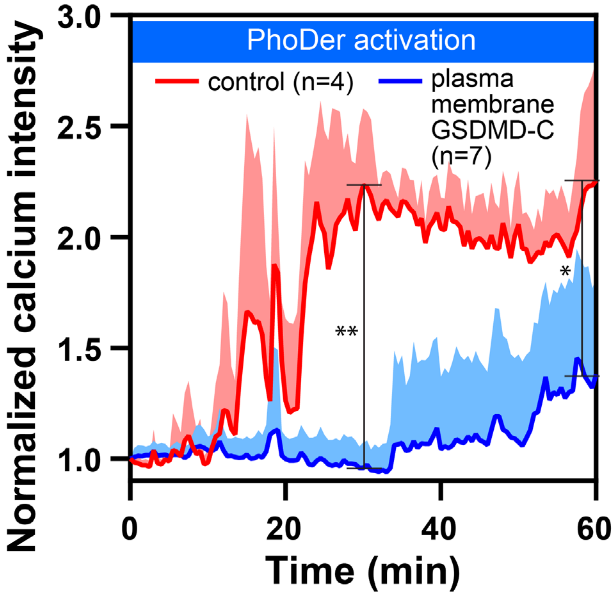

Supplementary Figure 4. Expression of the C-terminal domain of GSDMD (GSDMD-C) protects the cell after pyroptotic GSDMD activation. Optogenetically activated GSDMD does not require other protein partners to cause calcium response. Calcium response can be significantly inhibited by membrane localized (KRas CAAX) GSDMD-C domains (blue), despite the same optogenetic activation parameters as control (red), which lacks membrane GSDMD-C over-expression; line: mean, shaded: standard deviation. Significance from two-tailed Welch's t-test is p=0.0037 (**) at t=30 min and p=0.0376 (*) at t=60 minutes.

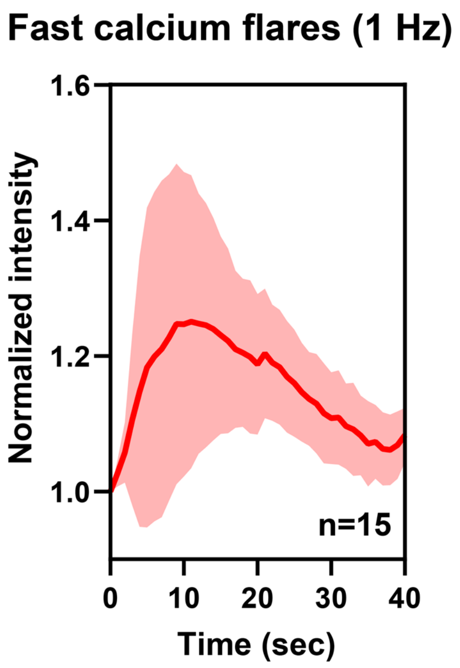

Supplementary Figure 5. Higher frequency calcium biosensing extends the upper speed limit of visible calcium flares. The averaged $t_{1/2}$ is approximately 3.5 seconds (over n=15 fastest peaks across n=5 cells); line: mean value, shaded region: standard deviation.

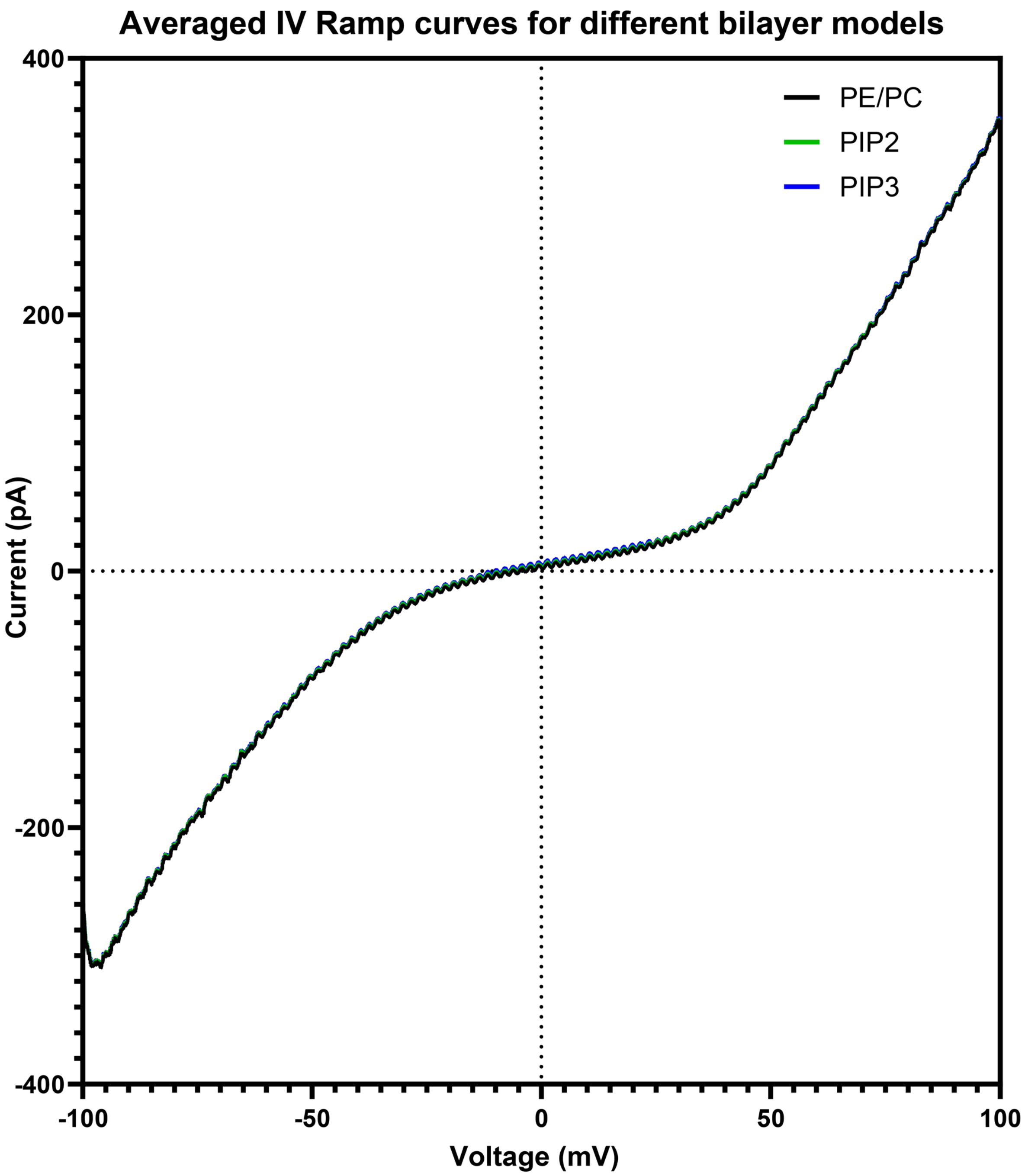

Supplementary Figure 6. Averaged I/V cycle curve demonstrating the dual-rectification property of GSDMD pores in bilayer models remains largely comparable whether in baseline PE/PC or with different additional phosphoinositides (PE/PC, n=9; PIP2 and PIP3, n=13 each).

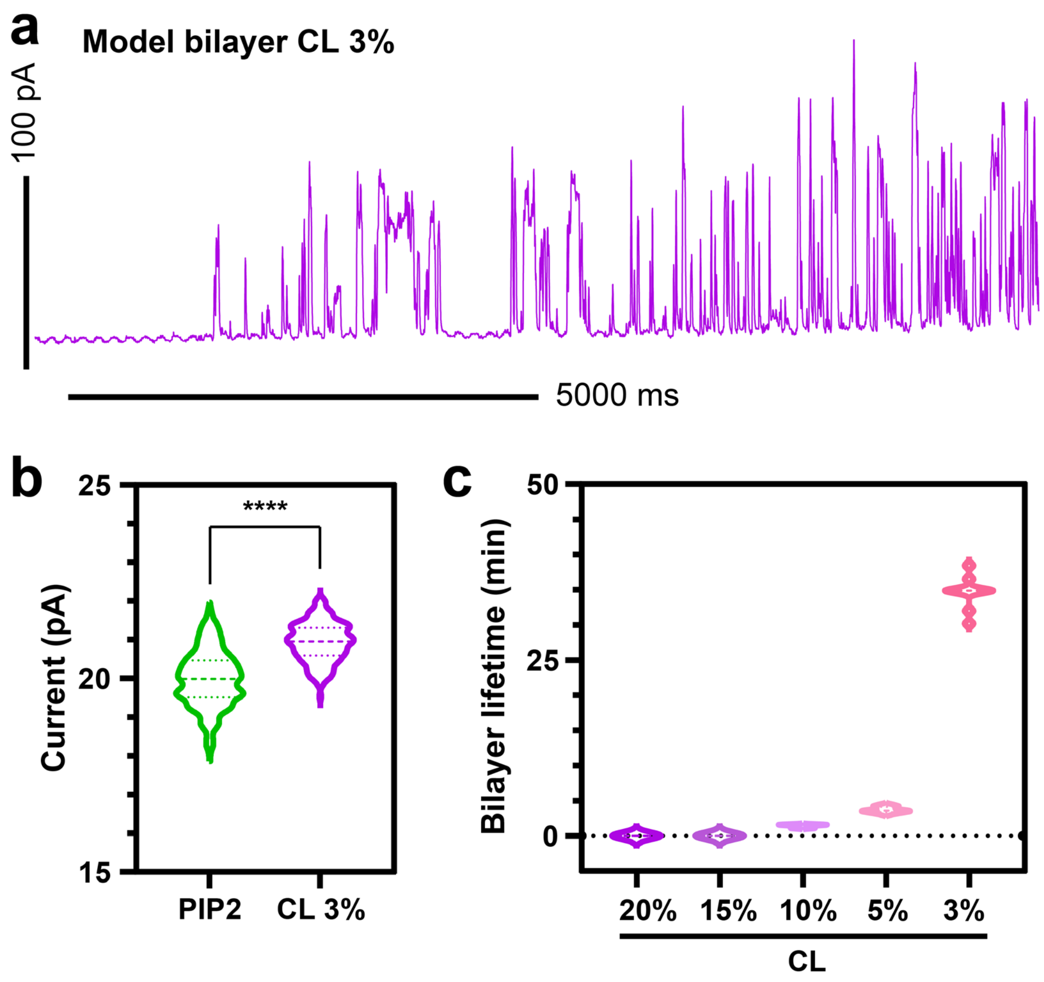

Supplementary Figure 7. The effect of cardiolipin (CL) on GSDMD pore dynamics is largely comparable to that of PIP2. (a) Representative single pore trace demonstrating that PE/PC bilayers containing 3% cardiolipin support reconstitution of GSDMD pores and display open/closing dynamics similar to phosphoinositide-containing bilayers. (b) Distribution of peak current from bilayers containing 3% CL, while statistically significant (****: $p<0.0001$, two-tailed Welch's t-test), is comparable in magnitude to that from bilayers containing PIP2. (c) CL has packing properties that destabilized these model bilayers at relative concentration of higher than 3% (n=10 attempts for each).

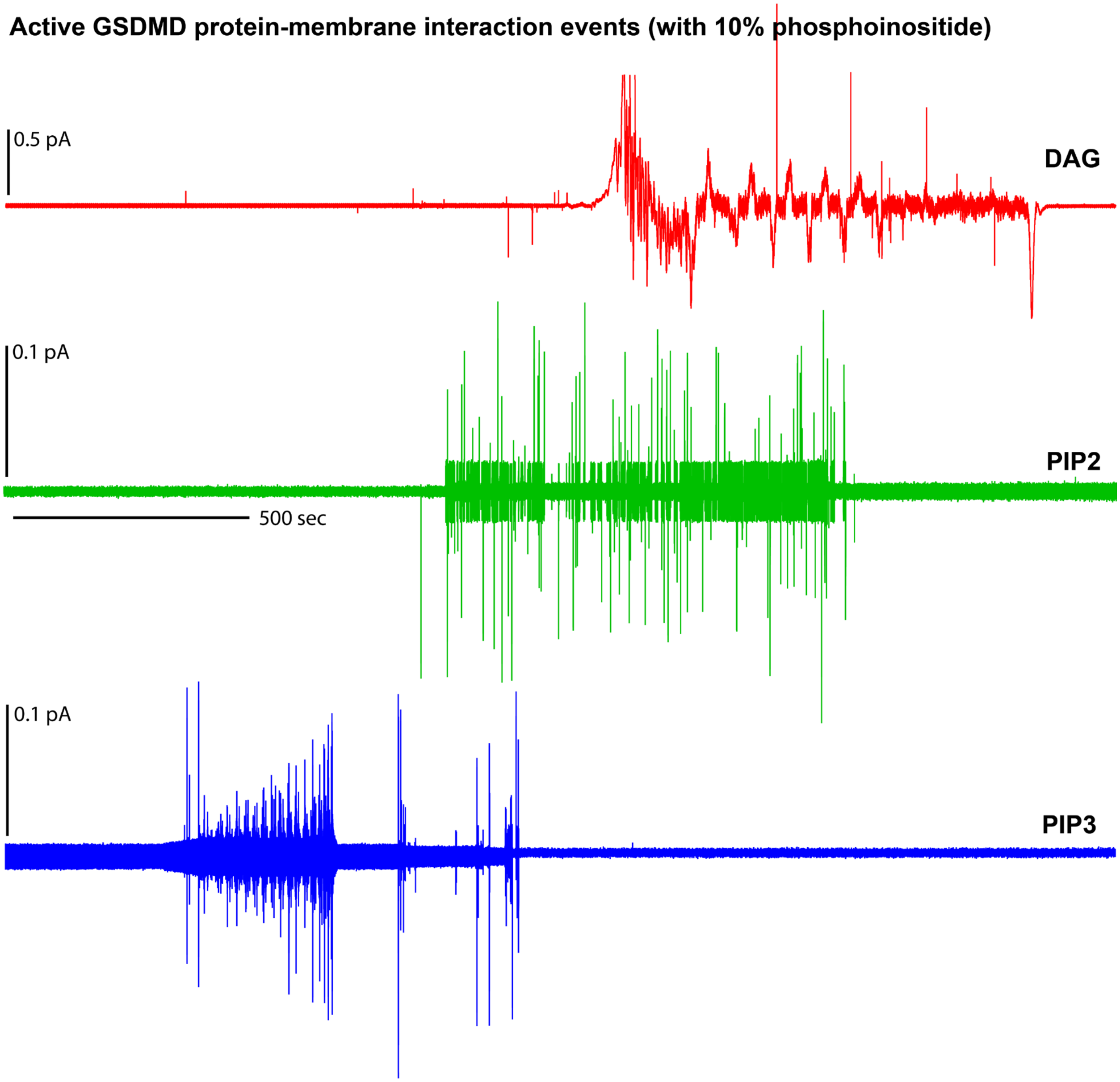

Supplementary Figure 8. Representative protein-membrane interaction micro-current events in phosphoinositide containing model bilayers. DAG: diacylglycerol; PIP2: PtdIns(4,5)P_2; and PIP3: PtdIns(3,4,5)P_3, respectively. The fatty acid tail lengths of these phosphoinositides were the same. While DAG bilayers showed micro-current events, no macroscopic currents could be measured unlike in bilayers with the other phosphoinositides PIP2 and PIP3.

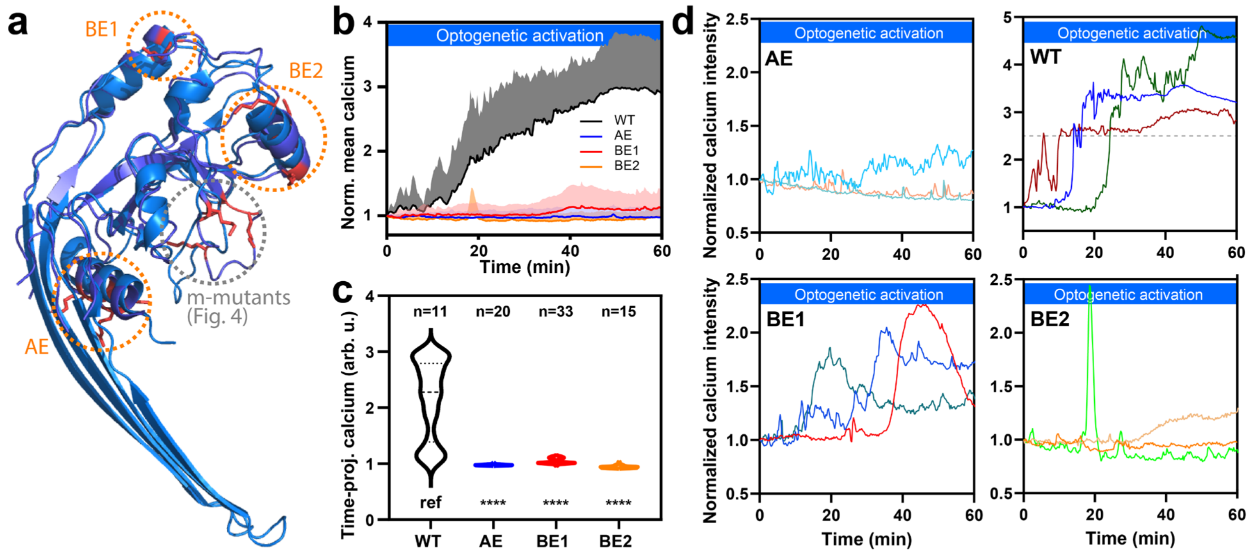

Supplementary Figure 9. Additional mutants in the structural exploration showing that mutations at additional lipid binding residues also impact GSDMD-calcium signaling. (a) Schematic for the additional regions targeted. Mutant labels: AE: R7E/10E/11E; BE1: R137E; BE2: R151E/R153E. For illustration, mGSDMA3 structure (PDB: 6CB8, blue) was aligned with the partial structure of the N-terminal domain of hGSDMD (PDB: 6N9O, purple-blue); positively charged target residues are highlighted (red) and location of the mutants reported in Figure. 4 are indicated for context. (b) Average whole cell calcium responses of the additional mutants were highly significantly inhibited compared to wild-type (WT) under the same optogenetic activation parameters; line: mean value; shaded region: standard deviation. (c) Time-projected average calcium response of the additional mutants. Statistical significance was reported from Two-way ANOVA with Tukey's multiple comparisons; "ref" represents the reference, ****: $p<0.0001$. (d) Representative calcium response in single HeLa cells demonstrating the effects of additional mutants; note that y-axes limit for the mutants is half that of WT and has been indicated by a dashed line in the WT plot for comparison.

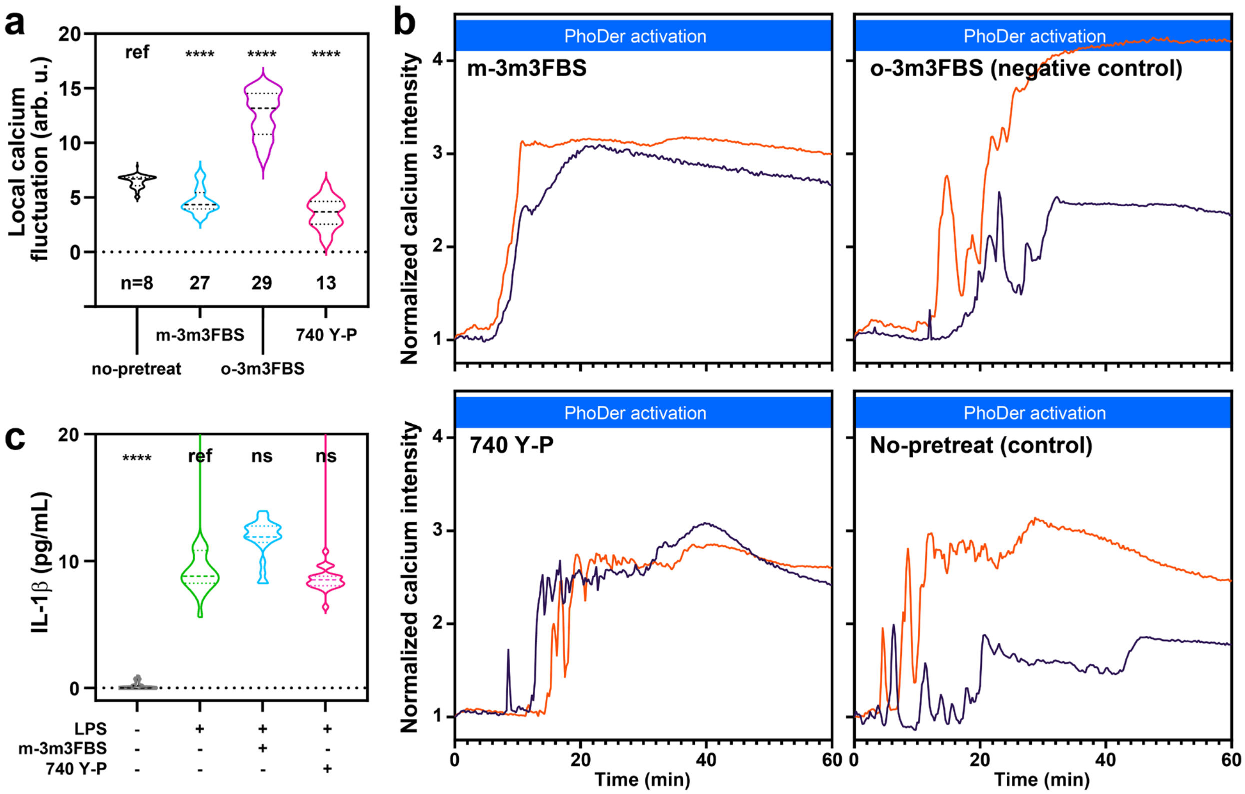

Supplementary Figure 10. Local GSDMD-calcium signaling feedback and downstream cytokine cannot be precisely modulated using PLC activator m-3m3FBS and PI3K activator 740 Y-P. (a) PLC activation using 50 µM m-3m3FBS and its cognate control (o-3m3FBS) showed decrease and increase in local calcium flare response, respectively, consistent with effects of PLC inhibition. PI3K activation using 50 µg/mL 740 Y-P showed a significant but slight decrease in calcium fluctuation. (b) Representative calcium response in single HeLa cells demonstrating the effects of activators; note that m-3m3FBS inhibited flares yet causes rapid increase in intracellular calcium, likely via a pathway independent to that studied here. (c) LPS induced IL-1β release in BMDM downstream of GSDMD activation was not significantly altered upon PLC activation or PI3K activation (8 technical replicate each for n=3 mouse). p-values in (a) and (c) are from two-tailed Welch's t-tests; ****: $p<0.0001$; ns: not significant.
