# Supplementary material for: Gasdermin D pores are dynamically regulated by local phosphoinositide circuitry
Source: Nat Commun. 2022 Jan 10;13:52. doi: 10.1038/s41467-021-27692-9 (PMC8748731; doi:10.1038/s41467-021-27692-9)
Supplement: Supplementary file 3 — Description of Additional Supplementary Files [file 41467_2021_27692_MOESM3_ESM.docx]

Description of Additional Supplementary Files

File name: Supplementary Movie 1

Description: Calcium progression during continuous activation of PhoDer in RAW264.7 macrophages expressing membrane targeted jRCaMP1b.

File name: Supplementary Movie 2

Description: Calcium progression during continuous activation of PhoDer in a bone-marrow derived macrophage (BMDM) expressing membrane targeted jRCaMP1b.

File name: Supplementary Movie 3

Description: Calcium progression during continuous activation of PhoDer in a HeLa cell expressing membrane targeted jRCaMP1b.
