# Supplementary material for: Gasdermin D pores are dynamically regulated by local phosphoinositide circuitry
Source: Nat Commun. 2022 Jan 10;13:52. doi: 10.1038/s41467-021-27692-9 (PMC8748731; doi:10.1038/s41467-021-27692-9)
Supplement: Supplementary file 7 — Reporting Summary [file 41467_2021_27692_MOESM7_ESM.pdf]

Corresponding author(s): Mo, Gary CH

Last updated by author(s): Nov 15, 2021

Reporting Summary

Nature Research wishes to improve the reproducibility of the work that we publish. This form provides structure for consistency and transparency in reporting. For further information on Nature Research policies, see our Editorial Policies and the Editorial Policy Checklist.

Statistics

For all statistical analyses, confirm that the following items are present in the figure legend, table legend, main text, or Methods section.

n/a	Confirmed	
☐	☒	The exact sample size (*n*) for each experimental group/condition, given as a discrete number and unit of measurement
☐	☒	A statement on whether measurements were taken from distinct samples or whether the same sample was measured repeatedly
☐	☒	The statistical test(s) used AND whether they are one- or two-sided<br>*Only common tests should be described solely by name; describe more complex techniques in the Methods section.*
☒	☐	A description of all covariates tested
☐	☒	A description of any assumptions or corrections, such as tests of normality and adjustment for multiple comparisons
☐	☒	A full description of the statistical parameters including central tendency (e.g. means) or other basic estimates (e.g. regression coefficient) AND variation (e.g. standard deviation) or associated estimates of uncertainty (e.g. confidence intervals)
☐	☒	For null hypothesis testing, the test statistic (e.g. F, t, r) with confidence intervals, effect sizes, degrees of freedom and P value noted<br>*Give P values as exact values whenever suitable.*
☒	☐	For Bayesian analysis, information on the choice of priors and Markov chain Monte Carlo settings
☒	☐	For hierarchical and complex designs, identification of the appropriate level for tests and full reporting of outcomes
☒	☐	Estimates of effect sizes (e.g. Cohen's d, Pearson's r), indicating how they were calculated

Our web collection on statistics for biologists contains articles on many of the points above.

Software and code

Policy information about availability of computer code

Data collection	All fluorescence images were collected using Nikon Elements software (ver. 5.11.01); all electrophysiology data were recorded using a Digidata 1440A recorder.
Data analysis	All fluorescence images were analyzed using a combination of standard and custom (for automation across the dataset) ImageJ plugins; all electrophysiology data were analyzed using Clampfit and R2. Analysis code was written for calcium flare imaging and automation in Matlab (2019a/b) or ImageJ (1.52v or newer). Statistical analyses were performed using GraphPad Prism (version 8.4 or newer). The code described has been deposited on GitHub under garymolab/2021_phoder.

For manuscripts utilizing custom algorithms or software that are central to the research but not yet described in published literature, software must be made available to editors and reviewers. We strongly encourage code deposition in a community repository (e.g. GitHub). See the Nature Research guidelines for submitting code & software for further information.

Data

Policy information about availability of data

All manuscripts must include a data availability statement. This statement should provide the following information, where applicable:

- Accession codes, unique identifiers, or web links for publicly available datasets
- A list of figures that have associated raw data
- A description of any restrictions on data availability

Associated raw (or "Source") data have been provided in spreadsheets named "Source Data" for main figures 1c/d, 2b/c, 3a-e, 4b-d, 5a-d, 6a-d; and supplementary figures 1a/c, 2a/b, 3a, 4, 5, 6, 7a-c, 8, 9b-d, 10a-c.

Field-specific reporting

Please select the one below that is the best fit for your research. If you are not sure, read the appropriate sections before making your selection.

☒ Life sciences ☐ Behavioural & social sciences ☐ Ecological, evolutionary & environmental sciences

For a reference copy of the document with all sections, see nature.com/documents/nr-reporting-summary-flat.pdf

Life sciences study design

All studies must disclose on these points even when the disclosure is negative.

Sample size	Sample size were maximized by available resources.
Data exclusions	All recorded cells and treated bone-marrow-derived macrophages were analyzed.
Replication	Data are reported from at least 3 biological and technical replicates. All replication attempts were successful.
Randomization	Mice studies were randomized; single cell studies were not explicitly randomized.
Blinding	Blinding was not applicable since designed constructs must be confirmed prior to experiments.

Reporting for specific materials, systems and methods

We require information from authors about some types of materials, experimental systems and methods used in many studies. Here, indicate whether each material, system or method listed is relevant to your study. If you are not sure if a list item applies to your research, read the appropriate section before selecting a response.

Materials & experimental systems

n/a	Involved in the study	
☒	☐	Antibodies
☐	☒	Eukaryotic cell lines
☒	☐	Palaeontology and archaeology
☐	☒	Animals and other organisms
☒	☐	Human research participants
☒	☐	Clinical data
☒	☐	Dual use research of concern

Methods

n/a	Involved in the study	
☒	☐	ChIP-seq
☒	☐	Flow cytometry
☒	☐	MRI-based neuroimaging

Eukaryotic cell lines

Policy information about cell lines

Cell line source(s)	RAW264.7 (ATCC); HeLa (ATCC)
Authentication	The cell lines utilized have not been authenticated
Mycoplasma contamination	Cell lines were monitored monthly using mycoplasma kit; all tests were negative
Commonly misidentified lines (See ICLAC register)	No commonly misidentified cell lines were used in the study

Animals and other organisms

Policy information about studies involving animals; ARRIVE guidelines recommended for reporting animal research

Laboratory animals	C57BL/6J mouse both female and male, aged 4-6 months
Wild animals	No wild animals were used in the study
Field-collected samples	No field-collected samples were used in the study
Ethics oversight	Office of Animal Care and Institutional Biosafety Committees, University of Illinois at Chicago

Note that full information on the approval of the study protocol must also be provided in the manuscript.
